# Tumor necrosis factor-alpha regulates photoreceptor cell autophagy after retinal detachment

**DOI:** 10.1038/s41598-017-17400-3

**Published:** 2017-12-07

**Authors:** Jia Xie, Ruilin Zhu, Yuan Peng, Wenna Gao, Jiantong Du, Liang Zhao, Ying Chi, Liu Yang

**Affiliations:** Department of Ophthalmology, Peking University First Hospital, No. 1 Xi’anmen Street, Xicheng District, Beijing, 100034 China

## Abstract

Photoreceptor cell death is the ultimate process underlying many retinal diseases, including retinal detachment (RD). Both autophagy and inflammatory factors, such as tumor necrosis factor-alpha (TNF-α), participate in photoreceptor cell death after RD. In this study, we examined whether TNF-α inhibition would impact the autophagy of photoreceptors and reduce the death of photoreceptors after retinal detachment (RD). RD models were created in C57BL/6J mice by a subretinal injection of 1% hyaluronic acid. The TNF-α inhibitor infliximab was administered via intraperitoneal injection two hours before RD. The levels of TNF-α and the autophagy-related proteins Atg5 and LC3B were assayed by immunofluorescence at 1 day, 3 days, and 7 days following RD. Apoptosis was examined at 3 days post-detachment via TUNEL assays. Photoreceptor cell counts were assessed at 7 days after RD. After RD, the protein levels of LC3B and Atg5 increased and reached a peak at 3 days, which decreased at 7 days. The expression of LC3B and Atg5 was prolonged and increased at a slower rate with TNF-α inhibition. The moderate augmentation and extension of autophagy through TNF-α inhibition resulted in the reduction of apoptosis and the enhancement of photoreceptor cell survival.

## Introduction

Photoreceptor cells play critical roles in the complex neural circuitry of the retina, which is responsible for transducing light signals into a pattern of electrical impulses. Unlike the non-mammalian vertebrates, which have a remarkable potential for retinal regeneration, mammals have very limited retinal regeneration^1,2^. Hence, the loss of photoreceptor cells always causes serious damage in mammals. In fact, photoreceptor cell death is the ultimate reason for irreversible visual impairment and blindness in a variety of retinal disorders, such as RD, retinitis pigmentosa (RP) and age-related macular degeneration (AMD), regardless of the great variety in the pathogenesis and clinical manifestation of these retinal diseases^3^. However, the processes of photoreceptor cell death in retinal diseases are still indistinct. Therefore, it is urgent that the molecular mechanisms involved in photoreceptor cell death and survival be elucidated.

RD, the separation of the neurosensory retina from the underlying retinal pigment epithelium (RPE), is one of the most sight-threatening diseases in ocular emergencies^4^. Photoreceptor cells are subjected to several insults when the physical separation occurs between photoreceptor cells and the RPE, which protects photoreceptor cells from light and oxide stimulation^5^. Experimentally induced RD is an appropriate model to study the mechanisms of photoreceptor cell death and rescue. The death of photoreceptor cells occurs immediately, as early as 12 h after RD, and peaks at approximately 2–3 days after RD both in human and experimental mouse models^6–8^. Apoptosis is the best researched form of cell death, and previous studies of photoreceptor cell death in last decade have always focused on apoptosis. However, anti-apoptosis treatment alone cannot completely prevent the death of photoreceptor cells. Accumulating evidence suggests that there are non-apoptosis pathways involved in photoreceptor cell death, such as autophagy^9^ and necrosis^10^.

Autophagy is one of the major forms of programmed cell death in the process of photoreceptor cell death that has attracted various attention. Pathologically, autophagy plays a complex role in photoreceptor cells, both protective and traumatic. Whether autophagy activity protects photoreceptor cells or facilitates the death of photoreceptor cells after RD is undefined. In recent years, as the exploration of autophagy has continued, researchers have found that inflammation plays a critical role in autophagy in other diseases; for example, the autophagy reaction introduced by TNF-α leads to the apoptosis of trophoblastic cells^11^. However, the influence of TNF-α on the autophagy of photoreceptor cells in a detached retina is ambiguous. Examining the relationship between autophagy and inflammation may provide a new perspective for the strategy of preventing photoreceptor cell loss in retinal degeneration diseases.

## Results

### TNF-α expression after RD

**Figure 1:**
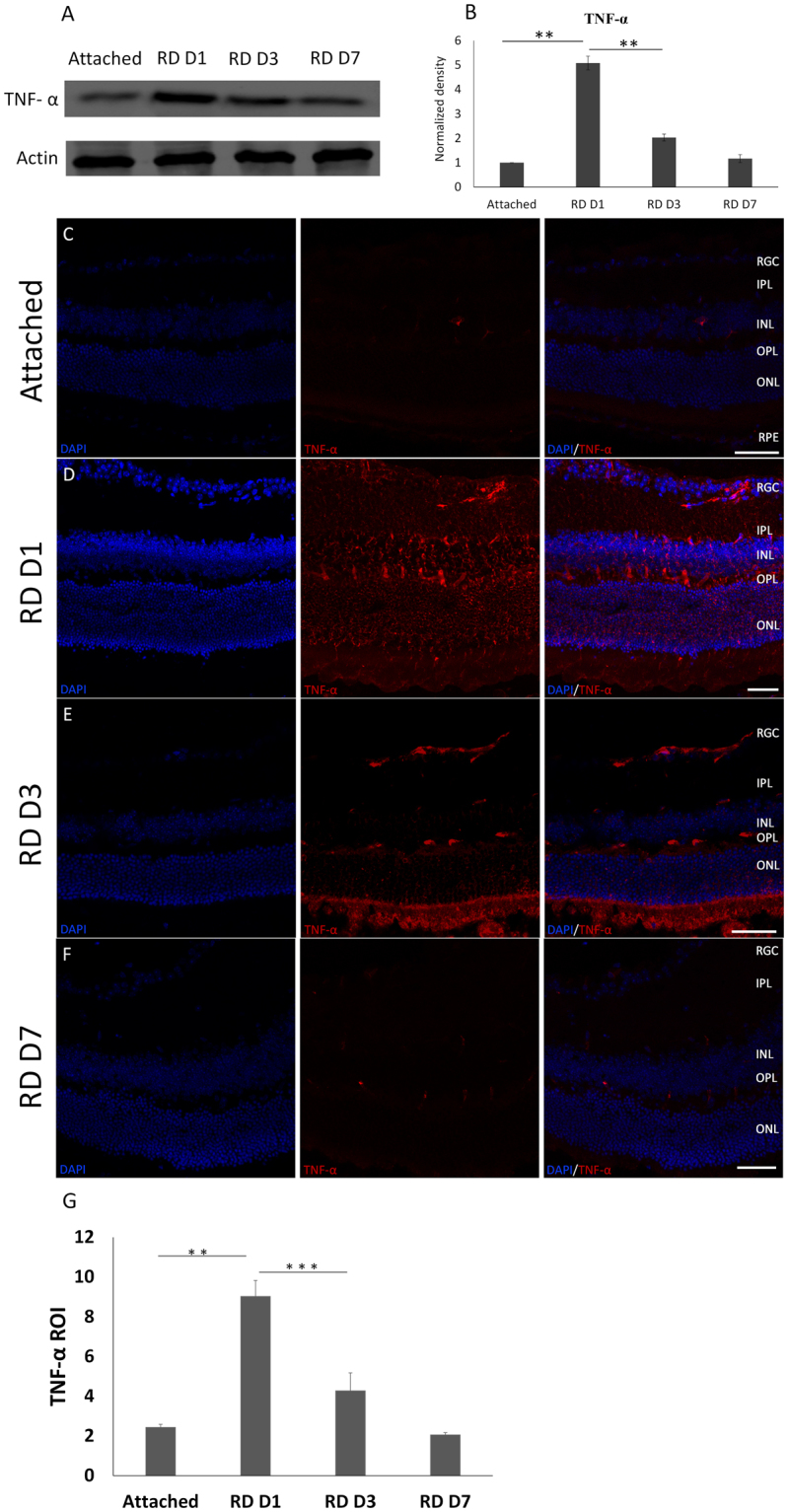
TNF-α activity after retinal detachment (RD) in mice. (**A**,**B**) Protein from attached and detached retinas was harvested at 1, 3, and 7 days after detachment and analyzed by Western blot. Attached retinas served as controls. TNF-α levels were peaked dramatically at 1 day and then decreased compared with attached retinas. Actin was used as a protein-loading control for all experiments. (**C**,**G**) TNF-α expression was analyzed by immunofluorescence in control retinas (**C**) and at 1 day (**D**), 3 days (**E**) and 7 days (**F**) after RD. (**G**) Three representative high-power fields of three separate eyes were measured in the ROI for photoreceptor cells. The expression of TNF-α was increased sharply at 1 day compared with the attached retinas. Error bars represent SD. All conditions were significantly different with *P < 0.05, **P < 0.01, and ***P < 0.005. GCL: ganglion cell layer; IPL: inner plexiform layer; INL: inner nuclear layer; OPL: outer plexiform layer; ONL: outer nuclear layer. (Scale bar: 50 μm).

To investigate the role of TNF-α in RD, the expression of TNF-α protein in the retina was detected at 1 day, 3 days and 7 days after RD by Western blotting and immunofluorescence. The Western blots showed that the TNF-α protein level was increased dramatically at 1 day following RD and then decreased (Fig. 1A and B). To confirm the variations in TNF-α in ONL where photoreceptor cells were located, immunofluorescence was performed. Consistent with the results for the total protein, the expression of TNF-α in retina peaked at 1 day, with a 4-fold increase in photoreceptor cells in the ONL and reduced to the baseline rates on the following days after RD (Fig. 1D–G), whereas the TNF-α immunoreactivity was very weak in controls (Fig. 1C). These data demonstrate an acute and intense increase in TNF-α in the photoreceptor cells after RD.

### Autophagy activity after RD

We examined two primary biochemical markers of autophagy, LC3B, which is an essential component of the autophagosome complex^12^ and Atg5, which is involved in the expansion of the autophagosome^13,14^. The expression of both LC3B and Atg5 in the retina after RD were examined by Western blotting.

**Figure 2:**
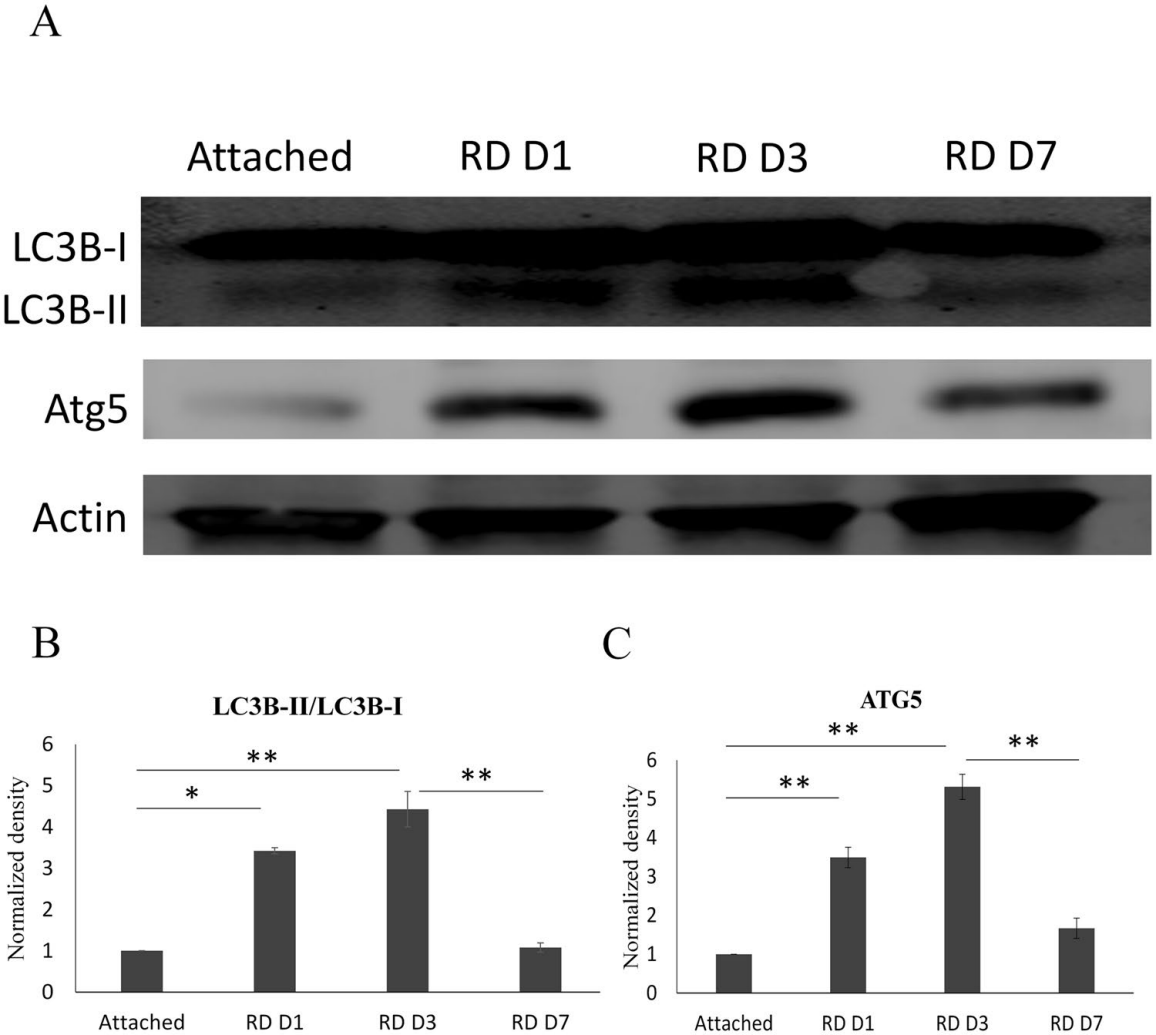
Autophagy activity after RD. (**A**) Western blot analysis of autophagy markers LC3B and Atg5. Whole retinas were harvested at 1, 3 and 7 days after RD. There was an increase in LC3B-II/LC3B-I (**B**) and Atg5 (**C**) levels at 1 and 3 days after RD compared to controls from attached retinas, and then a decline to the baseline at 7 days. Actin served as a loading control. Error bars represent SD. All conditions were significantly different with *P < 0.05, **P < 0.01, and ***P < 0.005.

LC3B migrates as two bands during polyacrylamide gel electrophoresis. When autophagy is activated, LC3B-I, which is an inactive form, would be transformed into LC3B-II, which is an essential marker of autophagy, indicating autophagosome formation^12^. The ratio of LC3B-II/LC3B-I expression was enhanced during 1 day to 3 days following RD (Fig. 2A and B) in accordance with prior results from others^15^. Similar to the variation tendency of LC3B, the total protein level of Atg5 in the retina was increased at 1 day to 3 days and decreased back to the baseline level at 7 days after RD (Fig. 2A and C).

### Blockade of TNF-α influences RD-induced autophagy

**Figure 3:**
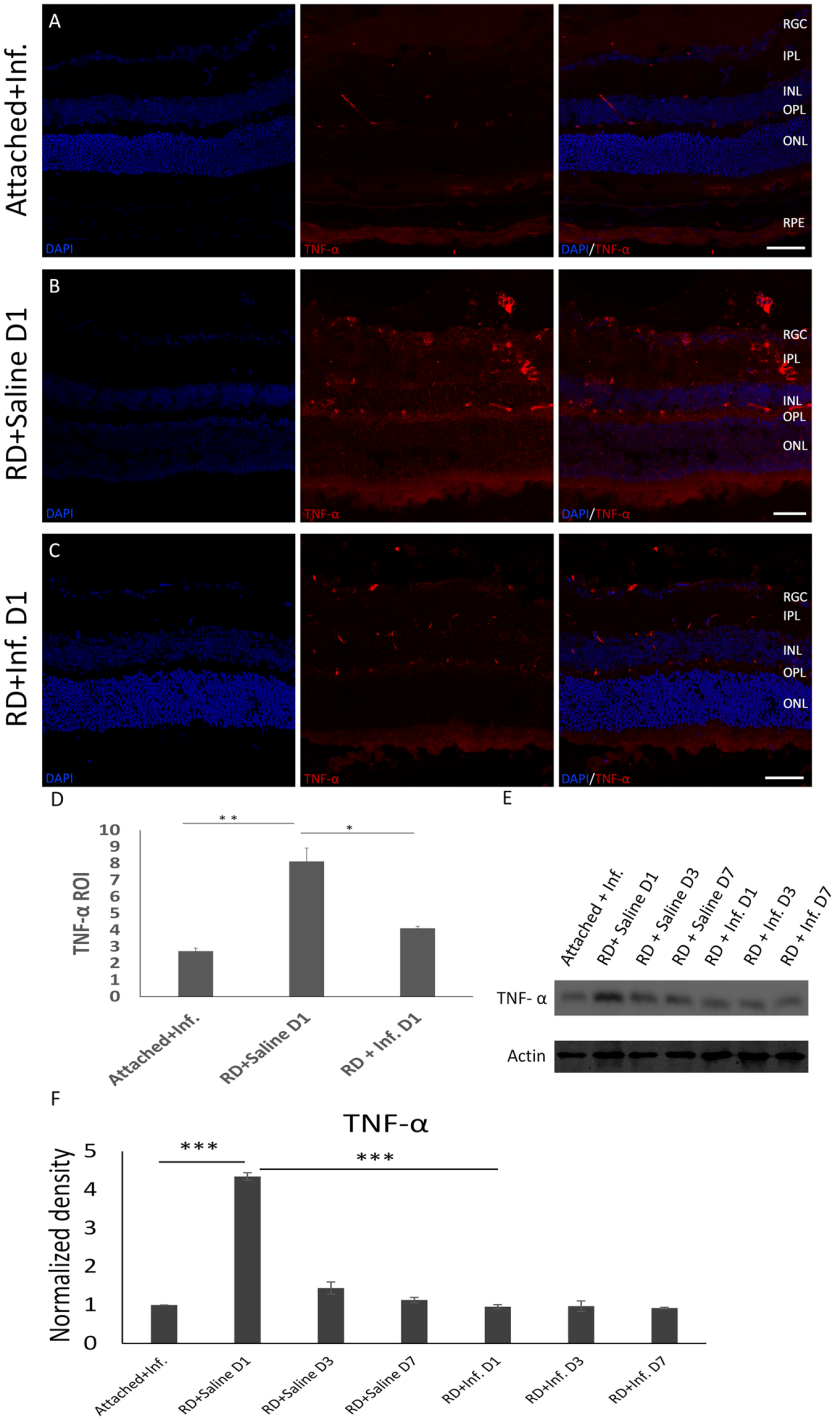
Infliximab inhibits TNF-α activity after RD. (**A**–**D**) Retinas were harvested and sectioned at 1 day following retinal detachment. Either 5 mg/kg infliximab or saline was injected intraperitoneally before detachment. Attached retinas served as controls (**A**). (**B**,**C**) The expression of TNF-α was suppressed after RD with TNF-α inhibition when compared with detached retinas at 1 day following RD without infliximab treatment. (**D**) The quantitative data for the ROIs in the TNF-α treatment group. Error bars represent SD. All conditions were significantly different with *P < 0.05 and **P < 0.01. (**E**,**F**) The protein level of TNF-α was analyzed by Western blot. The whole protein levels were obtained for TNF-α from attached retinas and detached retinas at 1, 3 and 7 days with or without infliximab treatment. TNF-α levels were inhibited at 1, 3 and 7 days after RD by infliximab. Actin served as a loading control. P-values were calculated using ANOVA analysis by SPSS20. The complete statistic results can be find in Supplementary Table 1. All conditions were significantly different with *P < 0.05, **P < 0.01, and ***P < 0.005.

To investigate whether TNF-α is involved in the regulation of RD-induced photoreceptor autophagy, a functional inhibitor of TNF-α infliximab (5 mg/kg) was intraperitoneally injected two hours before RD induction, and saline was as administered to controls. We quantified the levels of TNF-α by Immunofluorescence (Fig. 3A–D), Western blotting (Fig. 3E and F) at 1 day, 3 days and 7 days after RD. Infliximab successfully suppressed the expression of TNF-α in detached retina, especially in photoreceptor cells at 1 day after RD, while TNF-α levels peaked at 1 day following RD without TNF-α inhibition.

**Figure 4:**
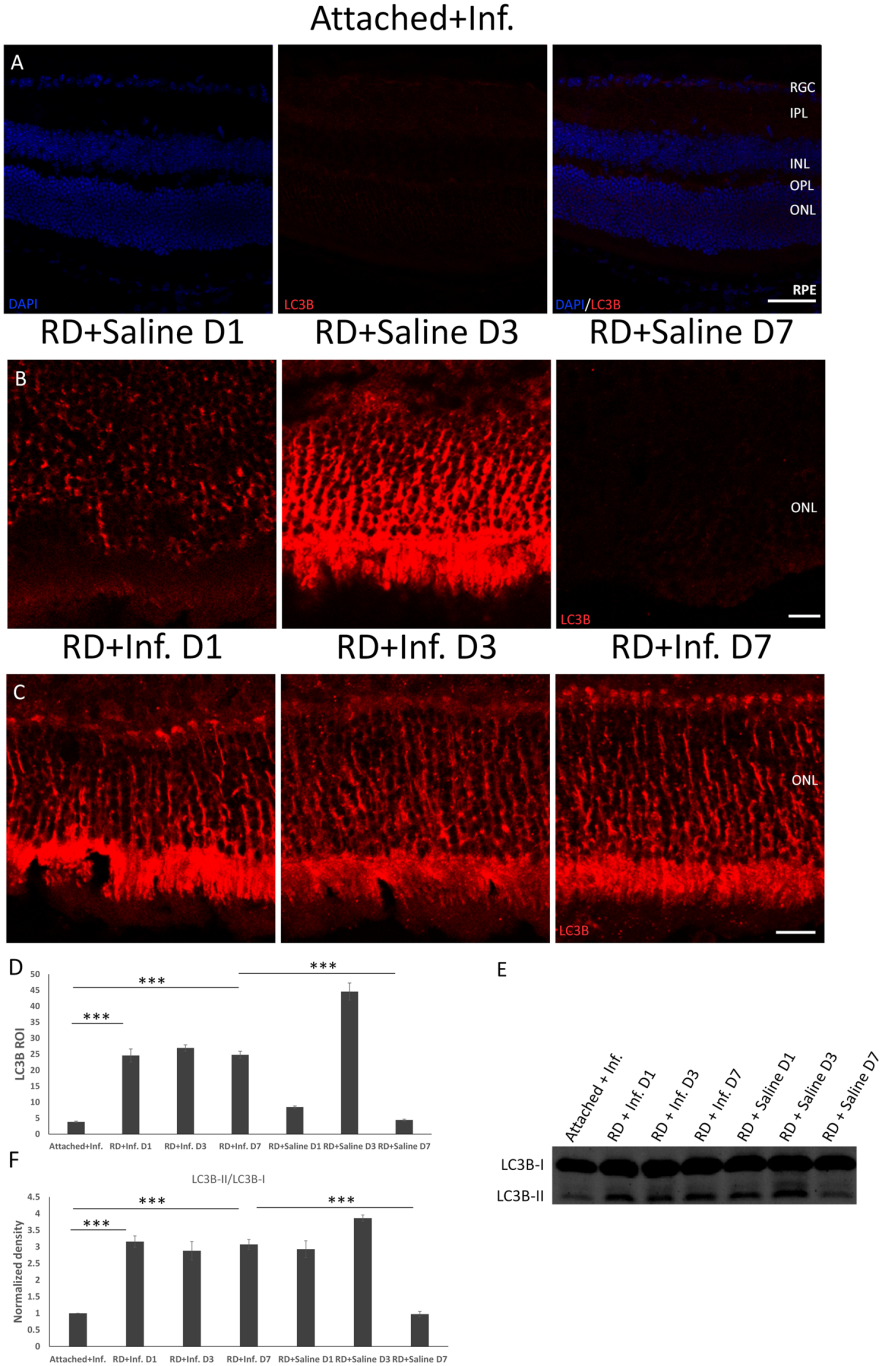
TNF-α inhibition influences LC3B activity after RD. (**A**–**D**) The levels of increased LC3B were prolonged and increased moderately at 1, 3 and 7 days after RD (**C**), compared with attached retinas (**A**) and detached retinas (**B**). (**D**) The quantitative data of the ROIs for LC3B expression after RD with TNF-α inhibition. Error bars represent SD. (**E**,**F**) The protein levels of LC3B analyzed by Western blot. With Infliximab treatment, the increased expression of LC3B-II and LC3B-II/LC3B-I was maintained through 7 days after RD, while the level of LC3B decreased to baseline at 7 days after RD without TNF-α inhibition. P-values were calculated using ANOVA analysis by SPSS20. The complete statistic results can be find in Supplementary Table 2 and Supplementary Table 3. All conditions were significantly different with *P < 0.05, **P < 0.01, and ***P < 0.005.

**Figure 5:**
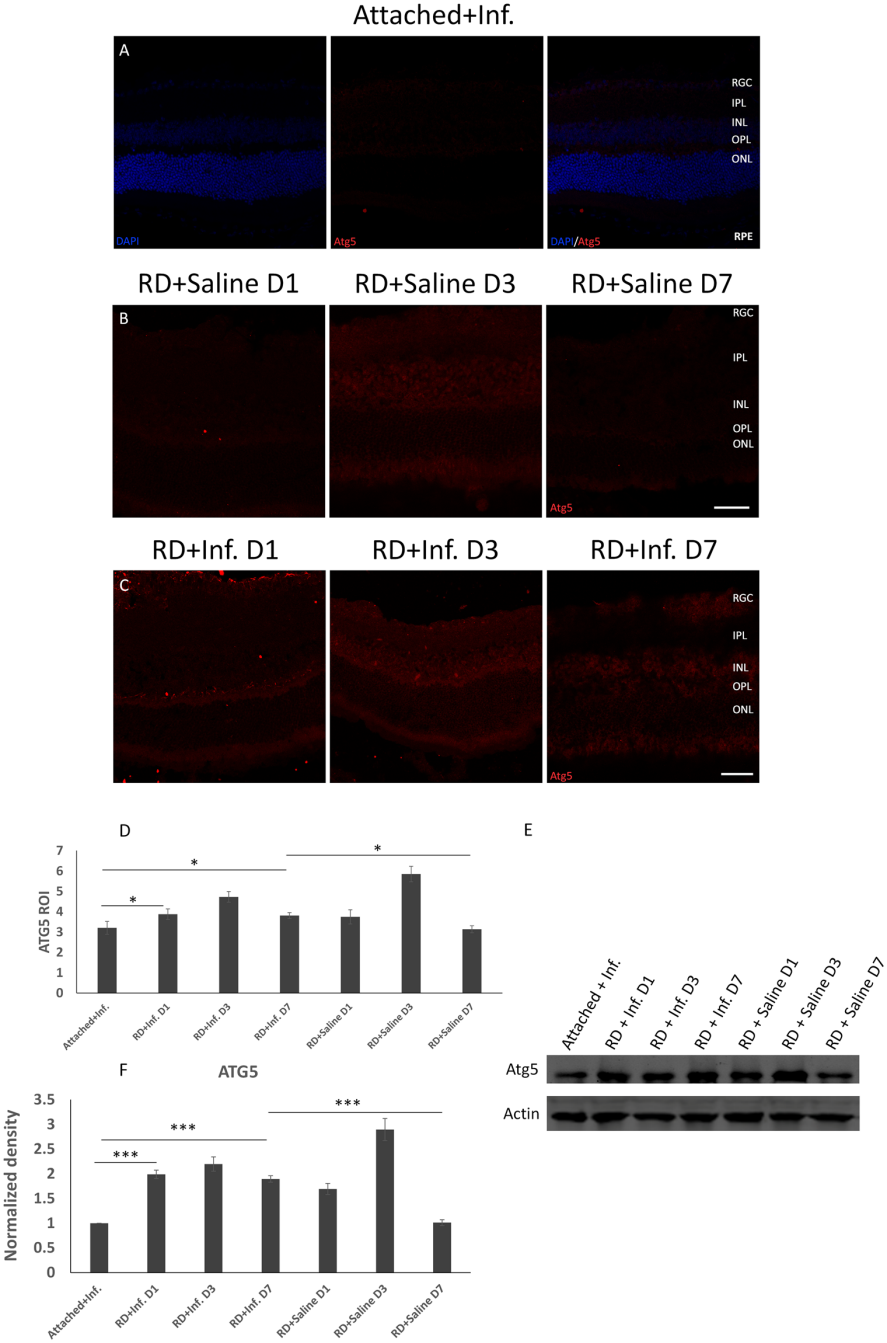
Atg5 activity is altered after RD with TNF-α blockade. (**A**–**D**) The levels of Atg5 after RD with TNF-α inhibition were analyzed by immunofluorescence. Compared with attached retinas with infliximab (**A**) and detached retinas added with saline (**B**), the expression of Atg5 was increased more moderately and was prolonged, as it was still enhanced at 7 days (**C**). (**D**) The quantitative data of the ROIs for Atg5 expression. Error bars represent SD. (**E**,**F**) The protein levels of Atg5 assayed by Western blot. Actin served as a loading control. P-values were calculated using ANOVA analysis by SPSS20. The complete statistic results can be find in Supplementary Table 4 and Supplementary Table 5. All conditions were significantly different with *P < 0.05, **P < 0.01, and ***P < 0.005.

Furthermore, we examined the levels of LC3B and Atg5 in detached retina after the reduction of TNF-α activity. Without infliximab treatment, LC3B was elevated at 1 day, peaked at 3 days with a 9-fold increase and declined to the baseline at 7 days after RD compared to controls (Fig. 4A,B and D). Interestingly, there was a persistently increased level of LC3B at 1 day, 3 days, and 7 days, whereas the degree of this increase was average and moderate with a 5-fold increase in expression in photoreceptor cells following RD with TNF-α inhibition compared with the attached retinas and detached retinas without the TNF-α blockade (Fig. 4C and D). Western blot analysis of LC3B also indicated that the increased expression of LC3B-II and the ratio of LC3B-II/LC3B-I maintained from 1 day to 7 days after RD (Fig. 4E and F). When the expression of Atg5 was examined in detached retina after blockade of TNF-α, there was an increase at 1 day to 3 days, which was similar to the tendency of LC3B in detached retina without infliximab treatment at 1 day to 3 days. However, the degree of its augmentation was eliminated compared to Atg5 levels in RD without infliximab treatment. What is more, the expression of Atg5 sustained enhancement at 7 days after RD with TNF-α inhibition, while Atg5 levels declined to the baseline at 7 days following RD without TNF-α inhibition (Fig. 5).

### The reduction of apoptosis and augmentation of cell survival

**Figure 6:**
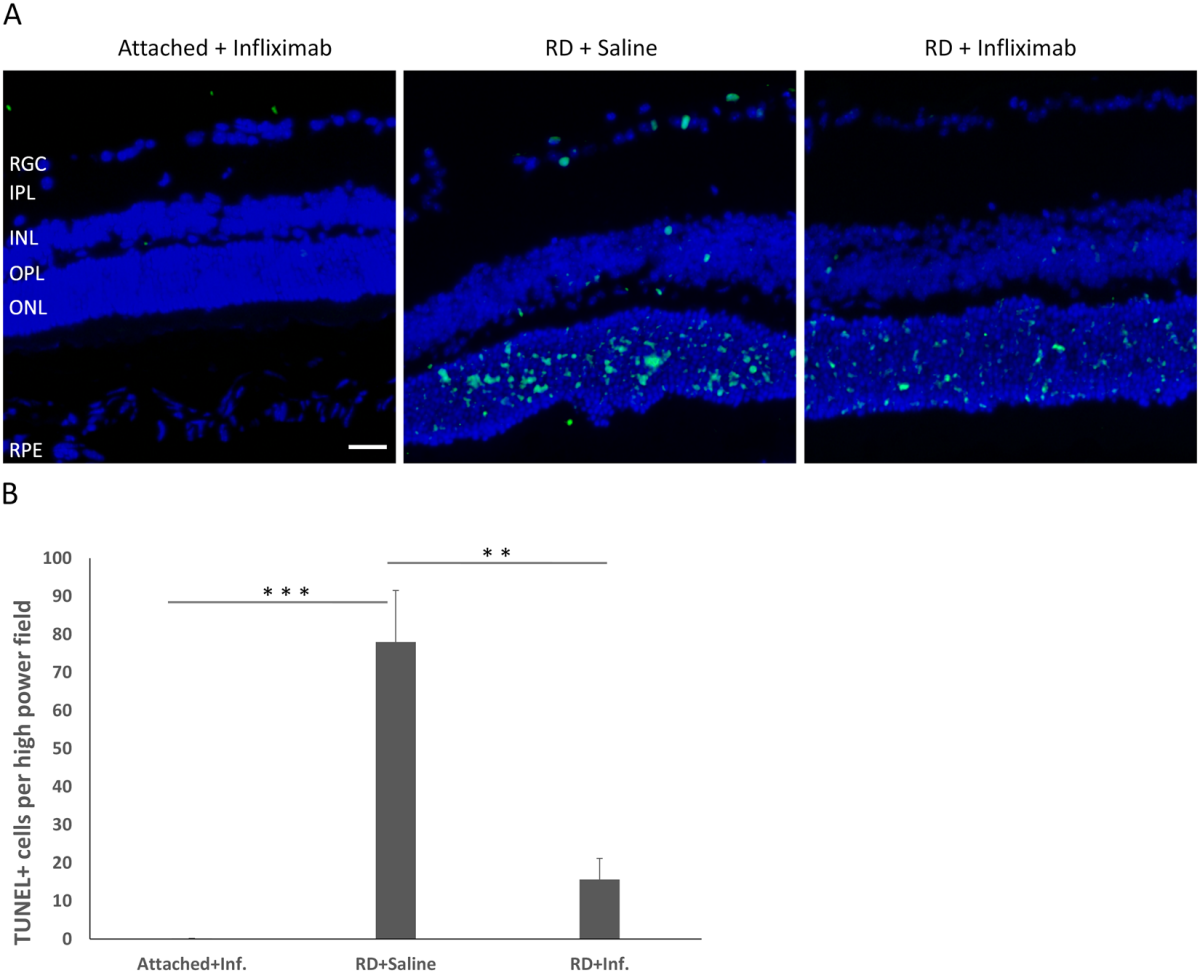
Reduction in apoptosis of photoreceptor cells after RD with TNF-α inhibition. (**A**) The retinas were harvested at 3 days after RD and photoreceptor apoptosis was analyzed by TUNEL. With TNF-α blockade, the number of TUNEL^+^ cells decreased dramatically, compared with retinas without infliximab treatment. (**B**) The quantitative data of TUNEL^+^ cells of attached retina, detached retina with or without infliximab treatment. Error bars represent SD. All conditions were significantly different at *P < 0.05, **P < 0.01, or ***P < 0.005.

**Figure 7:**
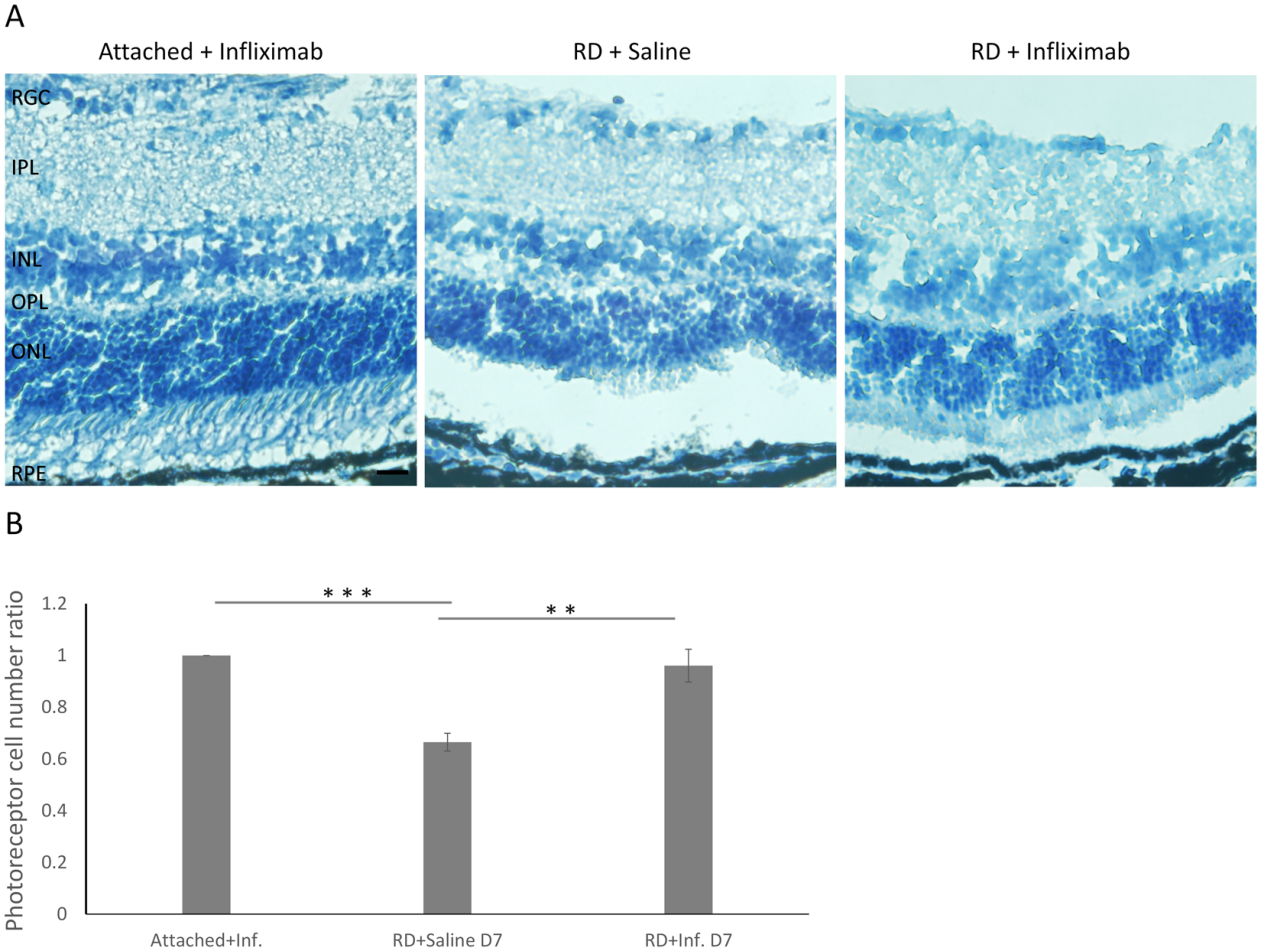
Improvement of photoreceptor cell survival following RD with TNF-α blockade. (**A**) The retinas were harvested at 7 days after RD. The photoreceptor cell number in the ONL decreased significantly, while the decline of photoreceptor cells was rescued with infliximab treatment. (**B**) Quantitation of the photoreceptor cell number ratios. Error bars represent SD. All conditions were significantly different at *P < 0.05, **P < 0.01, or ***P < 0.005.

Finally, to examine whether extending the autophagy caused by inhibiting TNF-α activity led to decreased apoptosis and increased cell survival in photoreceptors, we detected photoreceptor apoptosis using TUNEL at 3 days^7,16,17^ and cell survival by counting photoreceptor cells in the ONL at 7 days after inducing RD with infliximab treatment. Similar to a previous report that TUNEL^+^ cells peaked at 3 days following detachment^17^, we detected numerous TUNEL^+^ cells at 3 days after RD (Fig. 6). At 7 days following RD, the photoreceptor cell number in the ONL decreased substantially, with an approximately 40~50% loss^17^. However, in the presence of infliximab, there was a dramatic reduction of the appearance of TUNEL^+^ photoreceptor cells in the ONL (Fig. 6). At 7 days after RD, with infliximab treatment, there was not a decrease in the number of photoreceptor cells in the ONL of the retina (Fig. 7).

## Discussion

In this study, we showed that TNF-α was a critical regulator of photoreceptor autophagy that was correlated closely with the homeostasis of photoreceptor cells using an experimental RD model in mice. The expression of TNF-α levels increased rapidly in photoreceptor cells following RD. Blockade of TNF-α activity with its functional inhibitor, infliximab, prolonged RD-induced photoreceptor autophagy activity. Meanwhile, compared to detached retina without inhibition of TNF-α in which autophagy activity rises excessively, the increased autophagy maintained within more moderate levels after RD with infliximab treatment. Furthermore, the prolonged and temperate rise of autophagy activity introduced by inhibition of TNF-α expression lead to the elimination of photoreceptor apoptosis and the improvement of photoreceptor cell survival after RD.

Photoreceptor cells are highly specialized cells, and once the death of photoreceptor cells occurs, the loss can hardly be repaired^18^. To clarify the mechanisms of photoreceptor death, we created an experimental RD model in which photoreceptor cells were separated from the underlying RPE. The process of photoreceptor cell death has been thought to occur mainly by apoptosis. In our study, we examined the photoreceptor apoptosis at 3 days after RD by TUNEL. In accordance with previous research^17^, we detected an abundance of TUNEL^+^ cells at 3 days after RD. Although the death of photoreceptor cells can be delayed by the inhibition of apoptosis using pharmacological and genetic methods, the cytoprotective impact and rescue of the loss of photoreceptor cells are still limited. For illustration, caspases are activated in apoptotic photoreceptor cells; however, the inhibition of caspase in rodent eyes by intravitreal injection of the pan-caspase inhibitor Z-VAD fails to prevent the loss of photoreceptor cells following RD^10,19^. This suggests the participation of other processes, such as autophagy^20^ and necroptosis^21^. The relationship between autophagy and photoreceptor cell death has attracted intense attention in recent years. In our study, the inhibition of TNF-α expression led to a reduction of TUNEL^+^ cell appearance and an increase in photoreceptor cell survival in mice after RD, which was also shown by Nakazawa and coworkers in a rodent model of RD^22^. However, regulation of the activity of apoptosis only is insufficient for photoreceptor cell rescue, and other death pathways affected by TNF-α may cooperate with the caspase-dependent apoptosis and be involved in the death of photoreceptor cells. We hypothesized that the alteration of autophagy activity after RD would have a significant influence on photoreceptor cell death. In our research, we observed that autophagy in the form of programmed cell death is also regulated by TNF-α. Autophagy activity is prolonged and becomes more moderate, resulting in the decline of photoreceptor cell death and augmentation of photoreceptor cell survival after RD with TNF-α inhibitor treatment.

As a critical mechanism for maintaining intracellular homeostasis and quality control, autophagy plays a significant role in growth, adaptation, tumor suppression, aging, and innate and acquired immunity^18^. Recent studies indicate that autophagy is associated with the occurrence and development of many eye diseases, including corneal dystrophy^23^, cataracts^24^, glaucoma^25^ and retinal disorders such as age related macular degeneration^26^ and RD^20^. Our study clearly showed that the level of autophagy activity obviously varied after RD. Autophagy activity is increased at 1 day to 3 days and decreased to the baseline at 7 days after RD. However, autophagy plays a dual role in cell death and cell survival. Kunchithapautham and colleagues observed that marker genes of apoptosis and autophagy are co-expressed during the loss of photoreceptor cells that are exposed to oxidative stress; however, suppression of the activity of autophagy leads to a reduction of apoptosis, suggesting that autophagy takes part in the death of photoreceptor cells by triggering apoptosis^27^. Paradoxically, Besirli and colleagues report that inhibition of autophagy results in the augmentation of the expression of caspase 8 and an increase in the number of TUNEL^+^ photoreceptor cells after RD, suggesting a protective effect of autophagy activity^15^. In photoreceptor cells, whether autophagy activity plays a harmful or a protective role in photoreceptor cell demise or rescue during responses to injury remains controversial. We support the viewpoint that the influence of autophagy may be correlated with its degree. That is, initially, autophagy may play a pro-survival role, but this role becomes impaired resulting in cell death when the level of autophagy decreases or increases excessively. Chen and coworkers observed the activity of autophagy in ARPE19 cells incubated with 2.5 μM all-trans-retinal (atRAL) and found that cell death does not occur. However, the reduction of autophagy activity results in the appearance of cell death with increased atRAL treatment^28^. In our study, the level of autophagy was elevated excessively at 1 day to 3 days and declined to the baseline at 7 days following RD, which led to the loss of photoreceptor cells after RD, while the increase in autophagy became more moderate and protected the photoreceptor cells from death with the blockade of TNF-α activity, indicated by the reduction of TUNEL^+^ photoreceptor cells at 3 days. Furthermore, the protective autophagy activity became extended and did not decrease at 7 days with the use of the TNF-α inhibitor, resulting in the reduction of photoreceptor cell death and promotion of cell survival.

In conclusion, we demonstrated that TNF-α played a significant role in the regulation of autophagy activity after RD. Autophagy was thought to have a complex influence on cell death and cell survival. We proved that the appropriate level of autophagy activity had a critical influence on the augmentation of cell survival. Control of autophagy to a proper level under pathological conditions may provide a new therapeutic approach to treat photoreceptor degeneration in retinal disorders.

## Materials and Methods

### Experimental Model of RD

All animal experiments were performed in accordance with the Association for Research in Vision and Ophthalmology Statement for the Use of Animals in Ophthalmic and Vision Research, and the protocols were approved by Peking University. Retinal detachment was induced in adult male (8–12 weeks) C57BL/6J mice provided by the Riken animal center. Mice were anesthetized with 10% chloral hydrate, and pupils were dilated with a topical application of 0.5% tropicamide (Santen, Inc.) and 0.5% phenylephrine hydrochloride (Santen, Inc.). A scleral puncture was made at the supernasal equator of the right eyeball with a glass micropipette to lower intraocular pressure. A glass micropipette was then inserted into the subretinal space, and 1~2 μL of 1.4% sodium hyaluronate was injected between the retina and the RPE. Mice receiving scleral puncture without a sodium hyaluronate injection served as the controls. Two hours before RD, 5 mg/kg body weight infliximab (Cliag AG, Switzerland) or saline was intraperitoneally injected. RD was created in only the right eye of each animal, and the left eye served as a control.

### Western Blot Analysis

Retinas from RD and control eyes without detachment were dissected from the RPE–choroid, homogenized, lysed in RIPA lysis buffer, ultrasonicated on ice, and centrifuged at 12,000 rpm for 10 min at 4 °C. Retinas from three animals were pooled to create one sample for each individual experiment. The protein concentration of the supernatant was then determined using the Pierce BCA Protein Assay Kit (Thermo Scientific). The protein samples were separated using 15% SDS-PAGE. After electrophoretic separation, the proteins were blotted onto a nitrocellulose membrane using semi-dry electrophoretic transfer according to standard protocols (Bio-Rad). The primary antibodies used for WB were LC3B (Sigma, L7543, 1/1000), Atg5 (Abcam, ab78073, 1/200), TNF-α (Abcam, ab199013, 1/200), and actin (Proteintech, 60008–1, 1/2000). The secondary antibodies used were 800CW donkey anti-rabbit IgG, 800CW donkey anti-mouse IgG, 680LT donkey anti-rabbit IgG and 680LT donkey anti-mouse IgG (Odyssey, 926-32213, 926-32212, 926-68021, 926-68022). Membranes were scanned using an Odyssey Infrared Imaging System.

### Immunofluorescence

The eyes were harvested at defined times after RD. The animals were euthanized, and the eyes were enucleated. The whole eyes were fixed with 4% paraformaldehyde (PFA) solution in phosphate-buffered saline (PBS) for 24 h at 4 °C, dehydrated in 30% sucrose solution for 24 h at 4 °C, embedded in OCT at −20 °C, and sectioned at 10 μm using a Leica cryostat. Eye sections were fixed in 4% PFA/PBS for 10 min, washed with PBS twice and blocked for 1 h in 5% BSA in PBS containing 0.1% Triton-X (PBST) at room temperature. The primary antibodies were diluted in antibody dilution solution (1% BSA in PBST) in ratios from 1:100 to 1:1000 and used for incubation overnight at 4 °C. The secondary antibodies were diluted at 1:1000 in antibody dilution solution and applied for incubation for 2 h at room temperature. The primary antibodies used for immunofluorescence were LC3B (Cell Signaling Technology, 2775S, 1/1000), Atg5 (Abcam, ab78073, 1/100), and TNF-α (Abcam, ab199013, 1/100). The secondary antibodies used were Cy3 donkey anti-mouse IgG, Cy3 donkey anti-rabbit IgG (Jackson ImmunoResearch, 715-165-150, 711-165-152), Alexa488 donkey anti-mouse IgG, and Alexa488 donkey anti-rabbit IgG (Life Technologies, A16017, A16033). All images were obtained using a Zeiss LSM 780 confocal microscope. In each experiment, measurements were made on three eyes.

### Histology and TUNEL Staining

Eye sections were fixed with 4% PFA/PBS at 4 °C overnight. Samples were stained with 0.5% toluidine blue. TUNEL analysis was detected using an ApopTag Fluorescein Direct kit (Millipore, S7160) according to the manufacturer’s instructions. In each experiment, three non-overlapping high-power fields (40X) at the retinal detachment area were selected per section. The total number of cells and the number of TUNEL-positive cells in the outer nuclear layer (ONL) were measured and then averaged. For each experimental group, measurements were made on three eyes.

### Data Analysis

Adobe Photoshop was used to analyze the images. The software ImageJ was used for cell counting and region of interest (ROI) measurements. Statistical analysis comparing groups was performed using Student’s t-test, SPSS20. ANOVA analysis and Bonferroni correction as the post-hoc test following ANOVA. Probabilities of P < 0.05 were considered significant (*P < 0.05; **P < 0.01; ***P < 0.005).

## Electronic supplementary material

Supplementary table
