# Supplementary material for: Tumor necrosis factor-alpha regulates photoreceptor cell autophagy after retinal detachment
Source: Sci Rep. 2017 Dec 7;7:17108. doi: 10.1038/s41598-017-17400-3 (PMC5719449; doi:10.1038/s41598-017-17400-3)
Supplement: Supplementary file 1 — Supplementary table [file 41598_2017_17400_MOESM1_ESM.pdf]

# Tumor necrosis factor-alpha regulates photoreceptor cell autophagy after retinal detachment

Jia Xie[1], Ruilin Zhu[1], Yuan Peng[1], Wenna Gao[1], Jiantong Du[1], Liang Zhao[1], Ying Chi[1], Liu Yang[1]

1. Department of Ophthalmology, Peking University First Hospital, No. 1 Xi'anmen Street, Xicheng District, Beijing, 100034, China.

## Supplementary table 1

## TNF-α Normalized Density

P Value = 1.57488E-014 (ANOVA)

Bonferroni

| (I) Time | (J) Time | Mean value-different value (I-J) | Std. Error | Sig. | 95% Confidence Interval | |
|---|---|---|---|---|---|---|
| | | | | | Lower Bound | Upper Bound |
| Control | RD+Inf. Day1 | -3.3450667* | .0929979 | 7.121E-014 | -3.689087 | -3.001046 |
| | RD+Inf. Day3 | -.4408333* | .0929979 | 6.640E-003 | -.784854 | -.096813 |
| | RD+Inf. Day7 | -.1286667 | .0929979 | 1.000E+000 | -.472687 | .215354 |
| | RD+Saline Day1 | .0451333 | .0929979 | 1.000E+000 | -.298887 | .389154 |
| | RD+Saline Day3 | .0303267 | .0929979 | 1.000E+000 | -.313694 | .374347 |
| | RD+Saline Day7 | .0799000 | .0929979 | 1.000E+000 | -.264121 | .423921 |
| RD+Inf. Day1 | Control | 3.3450667* | .0929979 | 7.121E-014 | 3.001046 | 3.689087 |
| | RD+Inf. Day3 | 2.9042333* | .0929979 | 5.033E-013 | 2.560213 | 3.248254 |
| | RD+Inf. Day7 | 3.2164000* | .0929979 | 1.226E-013 | 2.872379 | 3.560421 |
| | RD+Saline Day1 | 3.3902000* | .0929979 | 5.914E-014 | 3.046179 | 3.734221 |
| | RD+Saline Day3 | 3.3753933* | .0929979 | 6.283E-014 | 3.031373 | 3.719414 |
| | RD+Saline Day7 | 3.4249667* | .0929979 | 5.133E-014 | 3.080946 | 3.768987 |
| RD+Inf. Day3 | Control | .4408333* | .0929979 | 6.640E-003 | .096813 | .784854 |
| | RD+Inf. Day1 | -2.9042333* | .0929979 | 5.033E-013 | -3.248254 | -2.560213 |
| | RD+Inf. Day7 | .3121667 | .0929979 | 9.872E-002 | -.031854 | .656187 |
| | RD+Saline Day1 | .4859667* | .0929979 | 2.697E-003 | .141946 | .829987 |
| | RD+Saline Day3 | .4711600* | .0929979 | 3.613E-003 | .127139 | .815181 |
| | RD+Saline Day7 | .5207333* | .0929979 | 1.376E-003 | .176713 | .864754 |
| RD+Inf. Day7 | Control | .1286667 | .0929979 | 1.000E+000 | -.215354 | .472687 |
| | RD+Inf. Day1 | -3.2164000* | .0929979 | 1.226E-013 | -3.560421 | -2.872379 |
| | RD+Inf. Day3 | -.3121667 | .0929979 | 9.872E-002 | -.656187 | .031854 |
| | RD+Saline Day1 | .1738000 | .0929979 | 1.000E+000 | -.170221 | .517821 |
| | RD+Saline Day3 | .1589933 | .0929979 | 1.000E+000 | -.185027 | .503014 |
| | RD+Saline Day7 | .2085667 | .0929979 | 8.741E-001 | -.135454 | .552587 |

| (I) Time | (J) Time | Mean value-different value (I-J) | Std. Error | Sig. | 95% Confidence Interval | |
|---|---|---|---|---|---|---|
| | | | | | Lower Bound | Upper Bound |
| RD+Saline Day1 | Control | -.0451333 | .0929979 | 1.000E+000 | -.389154 | .298887 |
| | RD+Inf. Day1 | -3.3902000* | .0929979 | 5.914E-014 | -3.734221 | -3.046179 |
| | RD+Inf. Day3 | -.4859667* | .0929979 | 2.697E-003 | -.829987 | -.141946 |
| | RD+Inf. Day7 | -.1738000 | .0929979 | 1.000E+000 | -.517821 | .170221 |
| | RD+Saline Day3 | -.0148067 | .0929979 | 1.000E+000 | -.358827 | .329214 |
| | RD+Saline Day7 | .0347667 | .0929979 | 1.000E+000 | -.309254 | .378787 |
| RD+Saline Day3 | Control | -.0303267 | .0929979 | 1.000E+000 | -.374347 | .313694 |
| | RD+Inf. Day1 | -3.3753933* | .0929979 | 6.283E-014 | -3.719414 | -3.031373 |
| | RD+Inf. Day3 | -.4711600* | .0929979 | 3.613E-003 | -.815181 | -.127139 |
| | RD+Inf. Day7 | -.1589933 | .0929979 | 1.000E+000 | -.503014 | .185027 |
| | RD+Saline Day1 | .0148067 | .0929979 | 1.000E+000 | -.329214 | .358827 |
| | RD+Saline Day7 | .0495733 | .0929979 | 1.000E+000 | -.294447 | .393594 |
| RD+Saline Day7 | Control | -.0799000 | .0929979 | 1.000E+000 | -.423921 | .264121 |
| | RD+Inf. Day1 | -3.4249667* | .0929979 | 5.133E-014 | -3.768987 | -3.080946 |
| | RD+Inf. Day3 | -.5207333* | .0929979 | 1.376E-003 | -.864754 | -.176713 |
| | RD+Inf. Day7 | -.2085667 | .0929979 | 8.741E-001 | -.552587 | .135454 |
| | RD+Saline Day1 | -.0347667 | .0929979 | 1.000E+000 | -.378787 | .309254 |
| | RD+Saline Day3 | -.0495733 | .0929979 | 1.000E+000 | -.393594 | .294447 |

# Supplementary table 2

## LC3B ROI

P Value = 4.476E-013 (ANOVA)

Bonferroni

| (I) Time | (J) Time | Mean value-different value (I-J) | Std. Error | Sig. | 95% Confidence Interval | |
|---|---|---|---|---|---|---|
| | | | | | Lower Bound | Upper Bound |
| Control | RD+Saline Day1 | -4.6270000 | 1.4106256 | 1.150E-001 | -9.845227 | .591227 |
| | RD+Saline Day3 | -40.7403333* | 1.4106256 | 1.480E-012 | -45.958560 | -35.522106 |
| | RD+Saline Day7 | -.5923333 | 1.4106256 | 1.000E+000 | -5.810560 | 4.625894 |
| | RD+INF+Day1 | -20.7670000* | 1.4106256 | 1.369E-008 | -25.985227 | -15.548773 |
| | RD+INF+Day3 | -23.1126667* | 1.4106256 | 3.305E-009 | -28.330894 | -17.894440 |
| | RD+INF+Day7 | -20.9863333* | 1.4106256 | 1.191E-008 | -26.204560 | -15.768106 |
| RD+Saline Day1 | Control | 4.6270000 | 1.4106256 | 1.150E-001 | -.591227 | 9.845227 |
| | RD+Saline Day3 | -36.1133333* | 1.4106256 | 7.770E-012 | -41.331560 | -30.895106 |
| | RD+Saline Day7 | 4.0346667 | 1.4106256 | 2.645E-001 | -1.183560 | 9.252894 |
| | RD+INF+Day1 | -16.1400000* | 1.4106256 | 3.611E-007 | -21.358227 | -10.921773 |
| | RD+INF+Day3 | -18.4856667* | 1.4106256 | 6.294E-008 | -23.703894 | -13.267440 |
| | RD+INF+Day7 | -16.3593333* | 1.4106256 | 3.041E-007 | -21.577560 | -11.141106 |
| RD+Saline Day3 | Control | 40.7403333* | 1.4106256 | 1.480E-012 | 35.522106 | 45.958560 |
| | RD+Saline Day1 | 36.1133333* | 1.4106256 | 7.770E-012 | 30.895106 | 41.331560 |
| | RD+Saline Day7 | 40.1480000* | 1.4106256 | 1.811E-012 | 34.929773 | 45.366227 |
| | RD+INF+Day1 | 19.9733333* | 1.4106256 | 2.287E-008 | 14.755106 | 25.191560 |
| | RD+INF+Day3 | 17.6276667* | 1.4106256 | 1.165E-007 | 12.409440 | 22.845894 |
| | RD+INF+Day7 | 19.7540000* | 1.4106256 | 2.644E-008 | 14.535773 | 24.972227 |
| RD+Saline Day7 | Control | .5923333 | 1.4106256 | 1.000E+000 | -4.625894 | 5.810560 |
| | RD+Saline Day1 | -4.0346667 | 1.4106256 | 2.645E-001 | -9.252894 | 1.183560 |
| | RD+Saline Day3 | -40.1480000* | 1.4106256 | 1.811E-012 | -45.366227 | -34.929773 |
| | RD+INF+Day1 | -20.1746667* | 1.4106256 | 2.004E-008 | -25.392894 | -14.956440 |
| | RD+INF+Day3 | -22.5203333* | 1.4106256 | 4.672E-009 | -27.738560 | -17.302106 |
| | RD+INF+Day7 | -20.3940000* | 1.4106256 | 1.738E-008 | -25.612227 | -15.175773 |
| RD+INF+Day1 | Control | 20.7670000* | 1.4106256 | 1.369E-008 | 15.548773 | 25.985227 |
| | RD+Saline Day1 | 16.1400000* | 1.4106256 | 3.611E-007 | 10.921773 | 21.358227 |
| | RD+Saline Day3 | -19.9733333* | 1.4106256 | 2.287E-008 | -25.191560 | -14.755106 |
| | RD+Saline Day7 | 20.1746667* | 1.4106256 | 2.004E-008 | 14.956440 | 25.392894 |
| | RD+INF+Day3 | -2.3456667 | 1.4106256 | 1.000E+000 | -7.563894 | 2.872560 |
| | RD+INF+Day7 | -.2193333 | 1.4106256 | 1.000E+000 | -5.437560 | 4.998894 |
| RD+INF+Day3 | Control | 23.1126667* | 1.4106256 | 3.305E-009 | 17.894440 | 28.330894 |
| | RD+Saline Day1 | 18.4856667* | 1.4106256 | 6.294E-008 | 13.267440 | 23.703894 |
| | RD+Saline Day3 | -17.6276667* | 1.4106256 | 1.165E-007 | -22.845894 | -12.409440 |

| | | | | | | |
|---|---|---|---|---|---|---|
| | RD+Saline Day7 | 22.5203333* | 1.4106256 | 4.672E-009 | 17.302106 | 27.738560 |
| | RD+INF+Day1 | 2.3456667 | 1.4106256 | 1.000E+000 | -2.872560 | 7.563894 |
| | RD+INF+Day7 | 2.1263333 | 1.4106256 | 1.000E+000 | -3.091894 | 7.344560 |
| RD+INF+Day7 | Control | 20.9863333* | 1.4106256 | 1.191E-008 | 15.768106 | 26.204560 |
| | RD+Saline Day1 | 16.3593333* | 1.4106256 | 3.041E-007 | 11.141106 | 21.577560 |
| | RD+Saline Day3 | -19.7540000* | 1.4106256 | 2.644E-008 | -24.972227 | -14.535773 |
| | RD+Saline Day7 | 20.3940000* | 1.4106256 | 1.738E-008 | 15.175773 | 25.612227 |
| | RD+INF+Day1 | .2193333 | 1.4106256 | 1.000E+000 | -4.998894 | 5.437560 |
| | RD+INF+Day3 | -2.1263333 | 1.4106256 | 1.000E+000 | -7.344560 | 3.091894 |

# Supplementary table 3

## LC3B-II/LC3B-I Normalized Density

P Value = 3.629E-010 (ANOVA)
Bonferroni

| (I) Time | (J) Time | Mean value-different value (I-J) | Std. Error | Sig. | 95% Confidence Interval | |
|---|---|---|---|---|---|---|
| | | | | | Lower Bound | Lower Bound |
| Control | RD+Inf. Day1 | -2.1569667* | .1725972 | 1.165E-007 | -2.795443 | -1.518490 |
| | RD+Inf. Day3 | -1.8811667* | .1725972 | 6.685E-007 | -2.519643 | -1.242690 |
| | RD+Inf. Day7 | -2.0708333* | .1725972 | 1.968E-007 | -2.709310 | -1.432357 |
| | RD+Saline Day1 | -1.9269667* | .1725972 | 4.932E-007 | -2.565443 | -1.288490 |
| | RD+Saline Day3 | -2.8640000* | .1725972 | 2.791E-009 | -3.502477 | -2.225523 |
| | RD+Saline Day7 | .0255333 | .1725972 | 1.000E+000 | -.612943 | .664010 |
| RD+Inf. Day1 | Control | 2.1569667* | .1725972 | 1.165E-007 | 1.518490 | 2.795443 |
| | RD+Inf. Day3 | .2758000 | .1725972 | 1.000E+000 | -.362677 | .914277 |
| | RD+Inf. Day7 | .0861333 | .1725972 | 1.000E+000 | -.552343 | .724610 |
| | RD+Saline Day1 | .2300000 | .1725972 | 1.000E+000 | -.408477 | .868477 |
| | RD+Saline Day3 | -.7070333* | .1725972 | 2.288E-002 | -1.345510 | -.068557 |
| | RD+Saline Day7 | 2.1825000* | .1725972 | 1.000E-007 | 1.544023 | 2.820977 |
| RD+Inf. Day3 | Control | 1.8811667* | .1725972 | 6.685E-007 | 1.242690 | 2.519643 |
| | RD+Inf. Day1 | -.2758000 | .1725972 | 1.000E+000 | -.914277 | .362677 |
| | RD+Inf. Day7 | -.1896667 | .1725972 | 1.000E+000 | -.828143 | .448810 |
| | RD+Saline Day1 | -.0458000 | .1725972 | 1.000E+000 | -.684277 | .592677 |
| | RD+Saline Day3 | -.9828333* | .1725972 | 1.163E-003 | -1.621310 | -.344357 |
| | RD+Saline Day7 | 1.9067000* | .1725972 | 5.638E-007 | 1.268223 | 2.545177 |
| RD+Inf. Day7 | Control | 2.0708333* | .1725972 | 1.968E-007 | 1.432357 | 2.709310 |
| | RD+Inf. Day1 | -.0861333 | .1725972 | 1.000E+000 | -.724610 | .552343 |
| | RD+Inf. Day3 | .1896667 | .1725972 | 1.000E+000 | -.448810 | .828143 |
| | RD+Saline Day1 | .1438667 | .1725972 | 1.000E+000 | -.494610 | .782343 |
| | RD+Saline Day3 | -.7931667* | .1725972 | 8.737E-003 | -1.431643 | -.154690 |
| | RD+Saline Day7 | 2.0963667* | .1725972 | 1.682E-007 | 1.457890 | 2.734843 |
| RD+Saline Day1 | Control | 1.9269667* | .1725972 | 4.932E-007 | 1.288490 | 2.565443 |
| | RD+Inf. Day1 | -.2300000 | .1725972 | 1.000E+000 | -.868477 | .408477 |
| | RD+Inf. Day3 | .0458000 | .1725972 | 1.000E+000 | -.592677 | .684277 |
| | RD+Inf. Day7 | -.1438667 | .1725972 | 1.000E+000 | -.782343 | .494610 |
| | RD+Saline Day3 | -.9370333* | .1725972 | 1.866E-003 | -1.575510 | -.298557 |
| | RD+Saline Day7 | 1.9525000* | .1725972 | 4.173E-007 | 1.314023 | 2.590977 |
| RD+Saline Day3 | Control | 2.8640000* | .1725972 | 2.791E-009 | 2.225523 | 3.502477 |
| | RD+Inf. Day1 | .7070333* | .1725972 | 2.288E-002 | .068557 | 1.345510 |
| | RD+Inf. Day3 | .9828333* | .1725972 | 1.163E-003 | .344357 | 1.621310 |

| | | | | | | |
|---|---|---|---|---|---|---|
| | RD+Inf. Day7 | .7931667* | .1725972 | 8.737E-003 | .154690 | 1.431643 |
| | RD+Saline Day1 | .9370333* | .1725972 | 1.866E-003 | .298557 | 1.575510 |
| | RD+Saline Day7 | 2.8895333* | .1725972 | 2.478E-009 | 2.251057 | 3.528010 |
| RD+Saline Day7 | Control | -.0255333 | .1725972 | 1.000E+000 | -.664010 | .612943 |
| | RD+Inf. Day1 | -2.1825000* | .1725972 | 1.000E-007 | -2.820977 | -1.544023 |
| | RD+Inf. Day3 | -1.9067000* | .1725972 | 5.638E-007 | -2.545177 | -1.268223 |
| | RD+Inf. Day7 | -2.0963667* | .1725972 | 1.682E-007 | -2.734843 | -1.457890 |
| | RD+Saline Day1 | -1.9525000* | .1725972 | 4.173E-007 | -2.590977 | -1.314023 |
| | RD+Saline Day3 | -2.8895333* | .1725972 | 2.478E-009 | -3.528010 | -2.251057 |

# Supplementary table 4

## ATG5 ROI

P Value = 4.947E-006 (ANOVA)
Bonferroni

| (I) Time | (J) Time | Mean value-different value (I-J) | Std. Error | Sig. | 95% Confidence Interval | |
|---|---|---|---|---|---|---|
| | | | | | Lower Bound | Lower Bound |
| Control | RD+Day1 | -.5393333 | .3057563 | 1.000E+000 | -1.670396 | .591729 |
| | RD+Day3 | -2.6433333* | .3057563 | 1.153E-005 | -3.774396 | -1.512271 |
| | RD+Day7 | .0716667 | .3057563 | 1.000E+000 | -1.059396 | 1.202729 |
| | RD+INF+Day1 | -.6713333 | .3057563 | 9.549E-001 | -1.802396 | .459729 |
| | RD+INF+Day3 | -1.5183333* | .3057563 | 4.352E-003 | -2.649396 | -.387271 |
| | RD+INF+Day7 | -.6003333 | .3057563 | 1.000E+000 | -1.731396 | .530729 |
| RD+Day1 | Control | .5393333 | .3057563 | 1.000E+000 | -.591729 | 1.670396 |
| | RD+Day3 | -2.1040000* | .3057563 | 1.585E-004 | -3.235062 | -.972938 |
| | RD+Day7 | .6110000 | .3057563 | 1.000E+000 | -.520062 | 1.742062 |
| | RD+INF+Day1 | -.1320000 | .3057563 | 1.000E+000 | -1.263062 | .999062 |
| | RD+INF+Day3 | -.9790000 | .3057563 | 1.343E-001 | -2.110062 | .152062 |
| | RD+INF+Day7 | -.0610000 | .3057563 | 1.000E+000 | -1.192062 | 1.070062 |
| RD+Day3 | Control | 2.6433333* | .3057563 | 1.153E-005 | 1.512271 | 3.774396 |
| | RD+Day1 | 2.1040000* | .3057563 | 1.585E-004 | .972938 | 3.235062 |
| | RD+Day7 | 2.7150000* | .3057563 | 8.368E-006 | 1.583938 | 3.846062 |
| | RD+INF+Day1 | 1.9720000* | .3057563 | 3.200E-004 | .840938 | 3.103062 |
| | RD+INF+Day3 | 1.1250000 | .3057563 | 5.200E-002 | -.006062 | 2.256062 |
| | RD+INF+Day7 | 2.0430000* | .3057563 | 2.186E-004 | .911938 | 3.174062 |
| RD+Day7 | Control | -.0716667 | .3057563 | 1.000E+000 | -1.202729 | 1.059396 |
| | RD+Day1 | -.6110000 | .3057563 | 1.000E+000 | -1.742062 | .520062 |
| | RD+Day3 | -2.7150000* | .3057563 | 8.368E-006 | -3.846062 | -1.583938 |
| | RD+INF+Day1 | -.7430000 | .3057563 | 6.120E-001 | -1.874062 | .388062 |
| | RD+INF+Day3 | -1.5900000* | .3057563 | 2.825E-003 | -2.721062 | -.458938 |
| | RD+INF+Day7 | -.6720000 | .3057563 | 9.510E-001 | -1.803062 | .459062 |
| RD+INF+Day1 | Control | .6713333 | .3057563 | 9.549E-001 | -.459729 | 1.802396 |
| | RD+Day1 | .1320000 | .3057563 | 1.000E+000 | -.999062 | 1.263062 |
| | RD+Day3 | -1.9720000* | .3057563 | 3.200E-004 | -3.103062 | -.840938 |
| | RD+Day7 | .7430000 | .3057563 | 6.120E-001 | -.388062 | 1.874062 |
| | RD+INF+Day3 | -.8470000 | .3057563 | 3.158E-001 | -1.978062 | .284062 |
| | RD+INF+Day7 | .0710000 | .3057563 | 1.000E+000 | -1.060062 | 1.202062 |
| RD+INF+Day3 | Control | 1.5183333* | .3057563 | 4.352E-003 | .387271 | 2.649396 |
| | RD+Day1 | .9790000 | .3057563 | 1.343E-001 | -.152062 | 2.110062 |
| | RD+Day3 | -1.1250000 | .3057563 | 5.200E-002 | -2.256062 | .006062 |

| | | | | | | |
|---|---|---|---|---|---|---|
| | RD+Day7 | 1.5900000* | .3057563 | 2.825E-003 | .458938 | 2.721062 |
| | RD+INF+Day1 | .8470000 | .3057563 | 3.158E-001 | -.284062 | 1.978062 |
| | RD+INF+Day7 | .9180000 | .3057563 | 1.996E-001 | -.213062 | 2.049062 |
| RD+INF+Day7 | Control | .6003333 | .3057563 | 1.000E+000 | -.530729 | 1.731396 |
| | RD+Day1 | .0610000 | .3057563 | 1.000E+000 | -1.070062 | 1.192062 |
| | RD+Day3 | -2.0430000* | .3057563 | 2.186E-004 | -3.174062 | -.911938 |
| | RD+Day7 | .6720000 | .3057563 | 9.510E-001 | -.459062 | 1.803062 |
| | RD+INF+Day1 | -.0710000 | .3057563 | 1.000E+000 | -1.202062 | 1.060062 |
| | RD+INF+Day3 | -.9180000 | .3057563 | 1.996E-001 | -2.049062 | .213062 |

# Supplementary table 5

## ATG5 Normalized Density

P Value = 2.424E-009 (ANOVA)

Bonferroni

| (I) Time | (J) Time | Mean value-different value (I-J) | Std. Error | Sig. | 95% Confidence Interval | |
|---|---|---|---|---|---|---|
| | | | | | Lower Bound | Lower Bound |
| Control | RD+Inf. Day1 | -.9884000* | .1185279 | 1.768E-005 | -1.426862 | -.549938 |
| | RD+Inf. Day3 | -1.1958000* | .1185279 | 1.758E-006 | -1.634262 | -.757338 |
| | RD+Inf. Day7 | -.8951667* | .1185279 | 5.585E-005 | -1.333628 | -.456705 |
| | RD+Saline Day1 | -.6926000* | .1185279 | 8.963E-004 | -1.131062 | -.254138 |
| | RD+Saline Day3 | -1.8930333* | .1185279 | 4.647E-009 | -2.331495 | -1.454572 |
| | RD+Saline Day7 | -.0116333 | .1185279 | 1.000E+000 | -.450095 | .426828 |
| RD+Inf. Day1 | Control | .9884000* | .1185279 | 1.768E-005 | .549938 | 1.426862 |
| | RD+Inf. Day3 | -.2074000 | .1185279 | 1.000E+000 | -.645862 | .231062 |
| | RD+Inf. Day7 | .0932333 | .1185279 | 1.000E+000 | -.345228 | .531695 |
| | RD+Saline Day1 | .2958000 | .1185279 | 5.393E-001 | -.142662 | .734262 |
| | RD+Saline Day3 | -.9046333* | .1185279 | 4.952E-005 | -1.343095 | -.466172 |
| | RD+Saline Day7 | .9767667* | .1185279 | 2.033E-005 | .538305 | 1.415228 |
| RD+Inf. Day3 | Control | 1.1958000* | .1185279 | 1.758E-006 | .757338 | 1.634262 |
| | RD+Inf. Day1 | .2074000 | .1185279 | 1.000E+000 | -.231062 | .645862 |
| | RD+Inf. Day7 | .3006333 | .1185279 | 4.984E-001 | -.137828 | .739095 |
| | RD+Saline Day1 | .5032000* | .1185279 | 1.712E-002 | .064738 | .941662 |
| | RD+Saline Day3 | -.6972333* | .1185279 | 8.374E-004 | -1.135695 | -.258772 |
| | RD+Saline Day7 | 1.1841667* | .1185279 | 1.985E-006 | .745705 | 1.622628 |
| RD+Inf. Day7 | Control | .8951667* | .1185279 | 5.585E-005 | .456705 | 1.333628 |
| | RD+Inf. Day1 | -.0932333 | .1185279 | 1.000E+000 | -.531695 | .345228 |
| | RD+Inf. Day3 | -.3006333 | .1185279 | 4.984E-001 | -.739095 | .137828 |
| | RD+Saline Day1 | .2025667 | .1185279 | 1.000E+000 | -.235895 | .641028 |
| | RD+Saline Day3 | -.9978667* | .1185279 | 1.580E-005 | -1.436328 | -.559405 |
| | RD+Saline Day7 | .8835333* | .1185279 | 6.481E-005 | .445072 | 1.321995 |
| RD+Saline Day1 | Control | .6926000* | .1185279 | 8.963E-004 | .254138 | 1.131062 |
| | RD+Inf. Day1 | -.2958000 | .1185279 | 5.393E-001 | -.734262 | .142662 |
| | RD+Inf. Day3 | -.5032000* | .1185279 | 1.712E-002 | -.941662 | -.064738 |
| | RD+Inf. Day7 | -.2025667 | .1185279 | 1.000E+000 | -.641028 | .235895 |
| | RD+Saline Day3 | -1.2004333* | .1185279 | 1.676E-006 | -1.638895 | -.761972 |
| | RD+Saline Day7 | .6809667* | .1185279 | 1.064E-003 | .242505 | 1.119428 |
| RD+Saline Day3 | Control | 1.8930333* | .1185279 | 4.647E-009 | 1.454572 | 2.331495 |
| | RD+Inf. Day1 | .9046333* | .1185279 | 4.952E-005 | .466172 | 1.343095 |
| | RD+Inf. Day3 | .6972333* | .1185279 | 8.374E-004 | .258772 | 1.135695 |
| | RD+Inf. Day7 | .9978667* | .1185279 | 1.580E-005 | .559405 | 1.436328 |

| | | | | | | |
|---|---|---|---|---|---|---|
| | RD+Saline Day1 | 1.2004333* | .1185279 | 1.676E-006 | .761972 | 1.638895 |
| | RD+Saline Day7 | 1.8814000* | .1185279 | 5.045E-009 | 1.442938 | 2.319862 |
| RD+Saline Day7 | Control | .0116333 | .1185279 | 1.000E+000 | -.426828 | .450095 |
| | RD+Inf. Day1 | -.9767667* | .1185279 | 2.033E-005 | -1.415228 | -.538305 |
| | RD+Inf. Day3 | -1.1841667* | .1185279 | 1.985E-006 | -1.622628 | -.745705 |
| | RD+Inf. Day7 | -.8835333* | .1185279 | 6.481E-005 | -1.321995 | -.445072 |
| | RD+Saline Day1 | -.6809667* | .1185279 | 1.064E-003 | -1.119428 | -.242505 |
| | RD+Saline Day3 | -1.8814000* | .1185279 | 5.045E-009 | -2.319862 | -1.442938 |
